# Evaluation of Biased and Balanced Salvinorin A Analogs in Preclinical Models of Pain

**DOI:** 10.3389/fnins.2020.00765

**Published:** 2020-07-21

**Authors:** Kelly F. Paton, Andrew Biggerstaff, Sophia Kaska, Rachel S. Crowley, Anne C. La Flamme, Thomas E. Prisinzano, Bronwyn M. Kivell

**Affiliations:** ^1^School of Biological Sciences, Centre for Biodiscovery, Faculty of Science, Victoria University of Wellington, Wellington, New Zealand; ^2^Department of Pharmaceutical Sciences, College of Pharmacy, University of Kentucky, Lexington, KY, United States; ^3^Department of Medicinal Chemistry, School of Pharmacy, The University of Kansas, Lawrence, KS, United States; ^4^Malaghan Institute of Medical Research, Wellington, New Zealand

**Keywords:** Salvinorin A, kappa opioid receptor, antinociception, biased agonism, anxiety

## Abstract

In the search for safer, non-addictive analgesics, kappa opioid receptor (KOPr) agonists are a potential target, as unlike mu-opioid analgesics, they do not have abuse potential. Salvinorin A (SalA) is a potent and selective KOPr agonist, however, clinical utility is limited by the short duration of action and aversive side effects. Biasing KOPr signaling toward G-protein activation has been highlighted as a key cellular mechanism to reduce the side effects of KOPr agonists. The present study investigated KOPr signaling bias and the acute antinociceptive effects and side effects of two novel analogs of SalA, 16-Bromo SalA and 16-Ethynyl SalA. 16-Bromo SalA showed G-protein signaling bias, whereas 16-Ethynyl SalA displayed balanced signaling properties. In the dose-response tail-withdrawal assay, SalA, 16-Ethynyl SalA and 16-Bromo SalA were more potent than the traditional KOPr agonist U50,488, and 16-Ethynyl SalA was more efficacious. 16-Ethynyl SalA and 16-Bromo SalA both had a longer duration of action in the warm water tail-withdrawal assay, and 16-Ethynyl had greater antinociceptive effect in the hot-plate assay, compared to SalA. In the intraplantar 2% formaldehyde test, 16-Ethynyl SalA and 16-Bromo SalA significantly reduced both nociceptive and inflammatory pain-related behaviors. Moreover, 16-Ethynyl SalA and 16-Bromo SalA had no anxiogenic effects in the marble burying task, and 16-Bromo SalA did not alter behavior in the elevated zero maze. Overall, 16-Ethynyl SalA significantly attenuated acute pain-related behaviors in multiple preclinical models, while the biased KOPr agonist, 16-Bromo SalA, displayed modest antinociceptive effects, and lacked anxiogenic effects.

## Introduction

Pain causes suffering and discomfort, often having profound effects on quality of life (Vartiainen et al., 2016). To treat pain, medicines are prescribed that typically activate the mu-opioid receptor (MOPr), such as morphine, codeine and fentanyl (Toblin et al., 2011). Unfortunately, chronic treatment with MOPr agonists can potentiate pain (Celerier et al., 2000; Roeckel et al., 2016), and lead to dependence and addiction (Compton and Volkow, 2006). Opioid overdoses are the leading cause of accidental death in the United States (Okie, 2010; Rudd et al., 2016). Additionally, rates of opioid overdose have also been rising globally (GBD 2017 Causes of Death Collaborators, 2018).

In contrast to MOPr agonists, kappa opioid receptor (KOPr) agonists play a critical role in regulating the reward system by contributing to the negative-feedback of dopamine (Di Chiara and Imperato, 1988). Studies have shown that acute administration of KOPr agonists show anti-addictive potential and have antinociceptive (Gallantine and Meert, 2008), anti-inflammatory (Binder et al., 2001; Bileviciute-Ljungar et al., 2006), antipruritic effects (Kumagai et al., 2010; Akiyama et al., 2015) and anticonvulsant and anti-seizure effects (Zangrandi et al., 2016). In contrast to MOPr agonists, KOPr agonists do not inhibit gastrointestinal transit (Porreca et al., 1984) or cause respiratory depression (Freye et al., 1983).

Despite these advantages, traditional KOPr agonists, such as U50,488, that have balanced signaling properties (Schattauer et al., 2012), are associated with many side effects including sedation, anxiety, aversion, and dysphoria limiting their clinical development (Mucha and Herz, 1985; Pfeiffer et al., 1986; Suzuki et al., 1992; Bals-Kubik et al., 1993; Privette and Terrian, 1995; Pande et al., 1996; Skoubis et al., 2001; Walsh et al., 2001; Mague et al., 2003; Kudryavtseva et al., 2004; Vunck et al., 2011; Ehrich et al., 2015; Zhang et al., 2015; Wang et al., 2016). The concept of biased agonism, whereby activation of a G-protein coupled receptor can result in differential activation of signal transduction pathways, suggests that it may be possible to separate desired physiological effects from adverse side effects. This highlights biased agonism as a potential characteristic that can be exploited for therapeutic advantage. It has been suggested that KOPr agonists that preferentially activate G-protein signaling pathways over β-arrestin recruitment are likely to have reduced side effects (Bruchas and Chavkin, 2010). In support of this concept, the only clinically available KOPr agonist, nalfurafine, has recently been shown to be extremely G-protein biased at the human KOPr (hKOPr) (Schattauer et al., 2017). However, finding evidence in support of G-protein biased agonism has proven more difficult than anticipated, and some studies have found that nalfurafine is a balanced agonist (Liu et al., 2019) or β-arrestin biased (Dunn et al., 2019). Unfortunately, nalfurafine failed to alleviate pain-related depression of operant behavior in rats (Lazenka et al., 2018), and failed in development as an analgesic candidate, instead, it is now clinically available as an antipruritic drug in Japan (Kumagai et al., 2010).

Salvinorin A (SalA) is a naturally occurring KOPr agonist (Roth et al., 2002; Chavkin et al., 2004) with proven antinociceptive and anti-inflammatory effects *in vivo* (Ansonoff et al., 2006; John et al., 2006; McCurdy et al., 2006; Aviello et al., 2011; Fichna et al., 2012; Guida et al., 2012; Simonson et al., 2015; Rossi et al., 2016). However, a short duration of action (Prisinzano, 2005; Butelman et al., 2009; Teksin et al., 2009; Ranganathan et al., 2012), with aversive (Zhang et al., 2005) and anxiogenic side effects (Braida et al., 2009), have limited the clinical development of SalA. Therefore, the structure of SalA has been altered in an attempt to develop G-protein biased KOPr agonists with fewer side effects (White et al., 2015; Kivell et al., 2018). Modifications have been made at the carbon-16 position on the furan ring of SalA, with the addition of an ethynyl group creating 16-Ethynyl SalA, and the addition of a bromine group creating 16-Bromo SalA (Riley et al., 2014). Promisingly, in rats, 16-Bromo SalA and 16-Ethynyl SalA attenuated drug-seeking in cocaine prime-induced reinstatement without decreasing spontaneous locomotor activity (Riley et al., 2014). To more fully understand the potential therapeutic effects of these SalA analogs and the involvement of KOPr signaling bias, the present study compared the cell signaling pathways of 16-Ethynyl SalA and 16-Bromo SalA and assessed their antinociceptive effects and side effect profiles in mice.

## Materials and Methods

### Drug Preparation

Salvinorin A was isolated and purified from *Salvia divinorum* leaves and assessed for purity (>98%) using high-performance liquid chromatography (HPLC). The SalA analogs, 16-Ethynyl SalA and 16-Bromo SalA, were synthesized as previously described (Riley et al., 2014) and were tested for purity (>95%) with HPLC. Nor-binaltorphimine (nor-BNI) and U50,488 were purchased from Sigma-Aldrich (St. Louis, MO, United States).

### Cellular Assays

The forskolin-induced cyclic adenosine monophosphate (cAMP) accumulation was measured in the HitHunter^TM^ assay in Chinese Hamster Ovary (CHO) cells stably expressing the hKOPr (DiscoverX Corporation, Fremont, CA, United States) as previously described (Riley et al., 2014). The β-arrestin2 recruitment assay was measured using the DiscoverX PathHunter^TM^ assay in U2OS cells stably expressing β-arrestin2 and hKOPr as previously described following the manufacturer’s protocol (Kivell et al., 2018). The cAMP accumulation data was normalized to the vehicle and forskolin-only controls. The β-arrestin2 recruitment data was normalized to the vehicle and U50,488 maximum response values. The following formula was used to calculate the bias factor as previously described (Crowley et al., 2016; Kivell et al., 2018), with U50,488 as the reference ligand:

$$\log\left(\mathrm{bias}\mathrm{factor}\right)=\log{\left(\frac{E_{\max\left(\mathrm{test}\right)}\times {\mathrm{EC}}_{50\,\left(\mathrm{control}\right)}}{{\mathrm{EC}}_{50\,\left(\mathrm{test}\right)}\times E_{max\,\left(control\right)}}\right)}_{G-\mathrm{protein}}-\log{\left(\frac{E_{\max\,\left(\mathrm{test}\right)}\times {\mathrm{EC}}_{50\,\left(\mathrm{control}\right)}}{{\mathrm{EC}}_{50\,\left(\mathrm{test}\right)}\times E_{\max\,\left(\mathrm{control}\right)}}\right)}_{\beta -a\,r\,r\,e\,s\,t\,i\,n\,2}$$

Using this formula, a bias factor of 1 is a balanced agonist relative to U50,488, less than 1 is a β-arrestin2 biased agonist and more than 1 is a G-protein biased agonist.

### Animals

Adult male B6-SJL (20–30 g, 8 + weeks old) were used for experimental procedures, except for the marble-burying and elevated zero maze procedures which used male C57Bl/6J mice. Animals were bred and housed at the Victoria University of Wellington Animal Facility, New Zealand, however, breeding stock animals were originally sourced from the Jackson Laboratories (Bar Harbor, ME, United States). The animals were group-housed (maximum five mice/cage) on a 12-h light/dark cycle (lights on at 07:00) with a stable temperature (19–21°C) and humidity (55%). All experimental procedures were carried out in the light cycle. Food and water were provided *ad libitum* except during experimental procedures. Animals were handled for at least 2 days before testing to reduce handling stress. All procedures were carried out with the approval of the Victoria University of Wellington Animal Ethics Committee (approval numbers 21480 and 25751). All procedures were carried out in agreement with the New Zealand Animal Welfare Act, 1999.

All KOPr agonists were dissolved in a vehicle containing DMSO, Tween-80 (Sigma-Aldrich) and physiological saline at a ratio of 2:1:7, respectively. The KOPr agonists were delivered at a volume of 10 μL/g of weight via intraperitoneal (i.p.) injection and delivered at 5 μL/g via subcutaneous (s.c.) injection in the warm water tail-withdrawal dose-response experiments. The KOPr antagonist nor-BNI was dissolved in physiological saline and injected s.c. 24 h prior to testing to selectively antagonize the KOPr, as earlier pre-treatment intervals have been shown to also antagonize the MOPr (Endoh et al., 1992; Kishioka et al., 2013).

### Warm Water Tail-Withdrawal Assay

On the 2 days prior to the test day, mice were restrained in transparent plastic tube restrainers (internal diameter 24 mm) for 5 min daily, to reduce restraint stress. Tail-withdrawal latencies were measured by immersing one-third of the distal portion of the tail in a warm water bath (50 ± 0.5°C) and the time to show the withdrawal response recorded. A maximum tail immersion cut-off of 10 s was used to avoid tissue damage and allow for repeated exposure to the thermal stimulus. On the day of the experiment, the baseline latency for each animal was measured by averaging three predrug tail-withdrawal latencies, with successive measurements taken at least 5 min apart. Following the experimental procedure, the percentage maximum possible effect (%MPE) was calculated for each animal at each time point by using the following formula:

$$\%\mathrm{MPE}=\left(\frac{\mathrm{test}\,\mathrm{latency}-\mathrm{baseline}\,\mathrm{latency}}{10-\mathrm{baseline}\,\mathrm{latency}}\right)\times 100$$

To measure the duration of action, the tail-withdrawal latencies were measured over a time-course as previously described (Simonson et al., 2015; Paton et al., 2017). Following the baseline latency measurements, mice were given an i.p. injection of either KOPr agonist or vehicle and the latency to tail-withdrawal behavior determined at 5, 10, 15, 30, 45, 60, 90, and 120 min for each animal in a repeated measures design.

### Cumulative Dose-Response Tail-Withdrawal Assay

The cumulative dose-response effects were evaluated using a within animal design, as previously described (Bohn et al., 2000; Paton et al., 2017; Sherwood et al., 2017). Briefly, mice were given s.c. injections at increasing concentrations to create cumulative doses in a volume of 5 μL/g of weight into the scruff of the neck, the left flank and the right flank in sequence to avoid continuous injections into the same area. At 30 min post-injection, the tail-withdrawal latency was measured and the next cumulative dose administered immediately after. The doses of 16-Ethynyl SalA and 16-Bromo SalA were delivered at: 0.3, 0.6, 1.0, 2.5, 5.0, 7.5, 10.0, and 12.5 mg/kg. Non-linear regression analysis was used to determine potency [median effective dose (ED_50_)] and efficacy calculated by normalizing data to the prototype KOPr agonist U50,488 [maximum dose achieved (E_*max*_)].

### Hot-Plate Assay

Mice were placed in a clear plastic enclosure positioned on a 50°C hot-plate (IITC Life Science, Woodland Hills, CA, United States). Timing began when all four paws were touching the hot-plate and stopped when mice exhibited any of the following behaviors: jumping or shaking/licking one of the hind paws. A cut-off time of 30 s was used to avoid tissue damage. The baseline latency was taken as the average of the three measurements, measured once per day for 3 days prior to testing. On the test day, an i.p. injection of the KOPr agonist or vehicle was administered and measurements were taken at 15, 30, and 60 min post-injection. The %MPE at each time point was calculated using the following formula:

$$\%\mathrm{MPE}=\left(\frac{\mathrm{test}\,\mathrm{latency}-\mathrm{baseline}\,\mathrm{latency}}{30-\mathrm{baseline}\,\mathrm{latency}}\right)\times 100$$

### Intraplantar 2% Formaldehyde Model

The Intraplantar 2% formaldehyde model was carried out as previously described (Paton et al., 2017; Sherwood et al., 2017). The custom-made chamber (27.5 × 18.5 cm) sat on a glass surface with a 45° angle mirror underneath to capture the behaviors via a video camera. The mice were habituated to the test enclosure for 15 min before testing. The mice were given KOPr agonist or vehicle treatment via i.p. injection 5 min prior to the administration of 20 μL of formaldehyde (2% in PBS) or PBS alone via intraplantar (i.pl.) injection. The animals were recorded for 60 min. The methods of Dubuisson and Dennis (1977) were used to assess the pain-like behavior using a weight-bearing score. The pain-related behavior was scored as 0 if the mouse showed normal behavior; (1) for partial weight-bearing with only the digits touching the floor; (2) with no weight-bearing with the paw raised; and (3) where the paw was bitten, licked or shaken. Scores were assessed every 5 s and averaged for every 5 min time period. For the area under the curve analysis, the two phases were separated out as follows: 0–15 min phase one nociceptive pain-related behavior; and 20–60 min phase two inflammatory pain-related behavior.

To test the local effects of the compounds, treatments were given as an i.pl. injection 5 min prior to and in the same hind paw as the 2% formaldehyde i.pl. injection. The KOPr agonist or vehicle were delivered at 1 μL/g with a maximum of 30 μL. The KOPr agonists were in a vehicle containing DMSO only.

### Rotarod Performance Assay

Mice were assessed for motor coordination on an accelerating rotarod apparatus with acceleration set from 4 to 40 rpm over 300 s (32 mm diameter barrel; Harvard Apparatus, Holliston, MA, United States) as previously described in Crowley et al. (2016). The mice were trained on the apparatus in a series of four sessions/day over 4 days and on test day, the latency to fall from the apparatus was measured, with a maximum of 300 s. On the test day, triplicate baseline measurements were taken and only mice with latencies for longer than 240 s in every trial were included in the experiment, to exclude any animals with pre-existing motor incoordination. The mice were administered an i.p. injection of either the KOPr agonist or vehicle and the latency to fall was measured repeatedly at 15, 30, 45, 60, 90, 120, 180, and 240 min. The results were expressed as a percentage of the individual animal’s baseline.

### Elevated Zero Maze

The elevated zero maze apparatus comprises a 5 cm wide elevated circular track with a 40 cm internal diameter and a 2 mm border. The maze is separated into four quadrants, two opposite quadrants are enclosed by 16 cm high dark, opaque walls, and two are open. Mice naïve to the maze were injected with either vehicle, SalA, 16-Ethynyl SalA or 16-Bromo SalA and 10 min later placed in the closed area of the apparatus. The mice were able to freely move within the apparatus for 5 min with behavior recorded from an overhead camera. The videos were assessed for time spent in the open arm, the number of open arm entries, and head dips over the edge of the open arm.

### Marble Burying

Clean cages were set up containing 5 cm of fresh bedding with 20 marbles arranged in rows. The mice were injected i.p. with either the KOPr agonist or vehicle and immediately placed in the cage and left undisturbed for 30 min. A photo was taken of each cage after the mouse was removed. Two observers who were blind to the treatment groups assessed the images for the number of marbles that remained unburied. Marbles were assigned as buried if less than a third of the marble was visible. Marbles were cleaned with 70% ethanol and autoclaved before being reused for subsequent mice.

### Data Analysis

GraphPad Prism (version 7.03, GraphPad Software, La Jolla, CA, United States) was used to determine statistical significance. Values represented as the mean ± standard error of the mean (SEM) and were considered significant when *p* < 0.05. The data sets were tested for normality using the D’Agostino and Pearson omnibus normality test. Unpaired *t*-tests were used to compare between two groups. Comparison of multiple treatment data was analyzed using one-way ANOVA followed by Bonferroni post-test (parametric) or Kruskal–Wallis followed by Dunn’s post-test (non-parametric). Comparisons of multiple effects over time were analyzed using two-way repeated-measures ANOVA followed by Bonferroni post-tests. The *p* values reported for the post-tests were adjusted for multiple families of comparisons.

## Results

**FIGURE 1:**
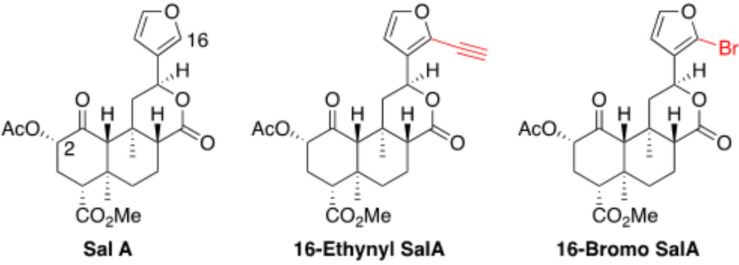
Chemical structures of Salvinorin A, 16-Ethynyl Salvinorin A, and 16-Bromo Salvinorin A.

**FIGURE 2:**
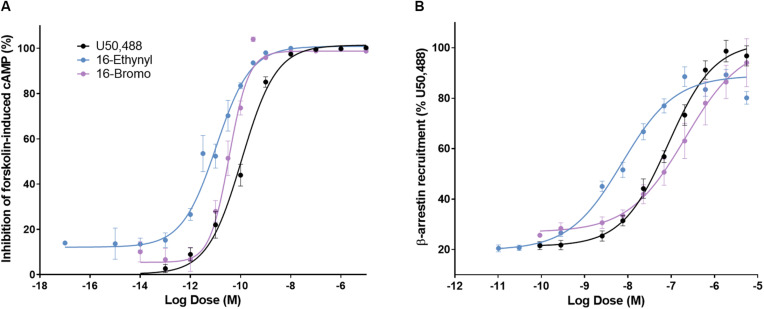
The activity of U50,488, 16-Ethynyl SalA, and 16-Bromo SalA in the cyclic adenosine monophosphate (cAMP) and β-arrestin recruitment assays. **(A)** The KOPr agonists were measured for inhibition of forskolin-induced cAMP (HitHunter^TM^): U50,488 *n* = 29 across 11 experiments, 16-Ethynyl SalA *n* = 36 across 16 experiments, 16-Bromo SalA *n* = 18 across six experiments and **(B)** β-arrestin recruitment (PathHunter^TM^): U50,488 *n* = 26 across nine experiments, 16-Ethynyl SalA *n* = 12 across three experiments, 16-Bromo SalA *n* = 15 across five experiments. Values presented as mean ± SEM.

A 16-Ethynyl SalA and 16-Bromo SalA are analogs of SalA with alterations at the C-16 position (Figure 1). To determine the bias factor of the compounds, both the G-protein and β-arrestin2 signaling pathways were measured. 16-Ethynyl SalA and 16-Bromo SalA had higher potency (EC_50_) than U50,488 at inhibiting forskolin-induced cAMP accumulation (Figure 2A and Table 1). The β-arrestin2 pathway was quantified using β-arrestin2 recruitment assays and showed that 16-Ethynyl SalA was more potent (EC_50_) than and 16-Bromo SalA and U50,488 (Figure 2B and Table 1). There were no significant differences in the efficacy (E_*max*_) between any of the compounds in these assays. The bias calculation revealed that 16-Bromo SalA was G-protein biased, whereas 16-Ethynyl SalA was a balanced agonist (Table 1).

**TABLE 1:** Functional activity and signaling bias of 16-Ethynyl SalA and 16-Bromo SalA in cAMP inhibition and β-arrestin recruitment assays.

	**Inhibition of forskolin-induced cAMP accumulation**			**β-arrestin2 recruitment**			
			
**Compound**	**EC_50_ (nM)**	**logEC_50_ ± SEM**	**E_max_ ± SEM**	**EC_50_ (nM)**	**logEC_50_ ± SEM**	**E_max_ ± SEM**	**Bias factor**
U50,488	0.111	−9.96 ± 0.073	100	84.9	−7.07 ± 0.084	100	1
16-Bromo SalA	0.035	−10.5 ± 0.059****	98.8 ± 1.84	212	−6.67 ± 0.298	102 ± 13.7	7.7 G
16-Ethynyl SalA	0.011	−11.0 ± 0.082****	101 ± 1.79	7.35	−8.13 ± 0.076***	89.0 ± 2.2	1.0

***p < 0.001, ****p < 0.0001 for comparisons to U50,488, analyzed using one-way ANOVA with Bonferroni post-tests.

**FIGURE 3:**
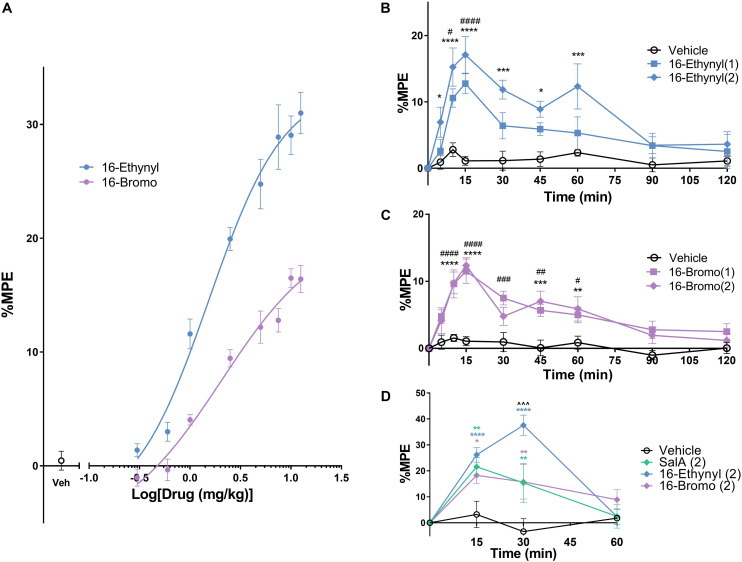
Antinociceptive effects of KOPr agonists in acute thermal pain-related behavioral models. **(A)** Cumulative dose-response effects of 16-Ethynyl SalA and 16-Bromo SalA in the warm water (50°C) tail-withdrawal assay. The maximal possible effect (%MPE) at each dose was calculated as a percentage based on the pre-treatment baseline latencies. Non-linear regression analysis showed 16-Ethynyl SalA and 16-Bromo SalA both exerted antinociceptive effects (*n* = 6). **(B,C)** The warm water (50°C) tail-withdrawal latencies were measured over a time course, up to 120 min (*n* = 7). **(B)** 16-Ethynyl SalA showed a significant effect up to 15 min for the 1 mg/kg dose and 60 min for the 2 mg/kg dose. **(C)** 16-Bromo SalA showed a significant duration of action for up to 60 min. **(D)** The %MPE of the paw withdrawal time on the hot-plate (50°C) was calculated based on pre-treatment baseline latencies (*n* = 6). Mice were treated with either vehicle, SalA (2 mg/kg), 16-Ethynyl SalA (2 mg/kg) or 16-Bromo SalA (2 mg/kg) and the withdrawal latencies measured up to 60 min. All compounds had a duration of action of 30 min, and 16-Ethynl SalA was more potent than SalA and 16-Bromo SalA at the 30 min time point. Two-way repeated-measures ANOVA followed by Bonferroni post-tests. *p < 0.05, **p < 0.01, ***p < 0.001, ****p < 0.0001 for 2 mg/kg doses vs. vehicle control. ^#^*p* < 0.05, ^##^*p* < 0.01, ^###^*p* < 0.001, ^####^*p* < 0.0001 for 1 mg/kg doses vs. vehicle control. ^∧∧∧^*p* < 0.001 for 16-Ethynyl SalA vs. both SalA and 16-Bromo SalA. Values presented as mean ± SEM.

The warm water tail-withdrawal assay measures the spinal reflex in response to a thermal stimulus. Non-linear regression analysis was used to determine the potency (median effective dose, ED_50_) and the efficacy (maximum response, E_*max*_, Figure 3A) and the values were compared to the traditional KOPr agonist, U50,488 (data set previously published in Paton et al., 2017). The ED_50_ values showed 16-Ethynyl SalA (ED_50_ = 1.54 mg/kg) and 16-Bromo SalA (ED_50_ = 2.06 mg/kg) were significantly more potent when compared to U50,488 (ED_50_ = 6.28 mg/kg). The E_*max*_ values showed that only 16-Ethynyl SalA (171.0 ± 21.5%) had a significantly higher maximum effect than U50,488.

To measure the onset and duration of action of the KOPr agonists, the warm water tail-withdrawal assay was used following a single i.p. injection, and the tail-withdrawal latencies measured over time. 16-Ethynyl SalA showed a significant antinociceptive effect for the 1 mg/kg dose at 10–15 min and a significant effect for the 2 mg/kg dose at 5–60 min (Figure 3B). 16-Bromo SalA showed significant antinociceptive effects at 1 mg/kg at 10–60 min and for the 2 mg/kg dose at 10–15 and 45–60 min (Figure 3C).

The hot-plate assay measures the withdrawal response from a thermal stimulus to the paws, which involves supraspinal processes. Similar to the previous experiment, the onset and duration of action can be measured using this technique. Bonferroni post-tests showed that SalA (2 mg/kg), 16-Bromo SalA and 16-Ethynyl SalA had a duration of action of 30 min (Figure 3D). In addition, at the 30 min time point, 16-Ethynyl SalA had a greater antinociceptive effect than both SalA and 16-Bromo SalA (Figure 3D).

**FIGURE 4:**
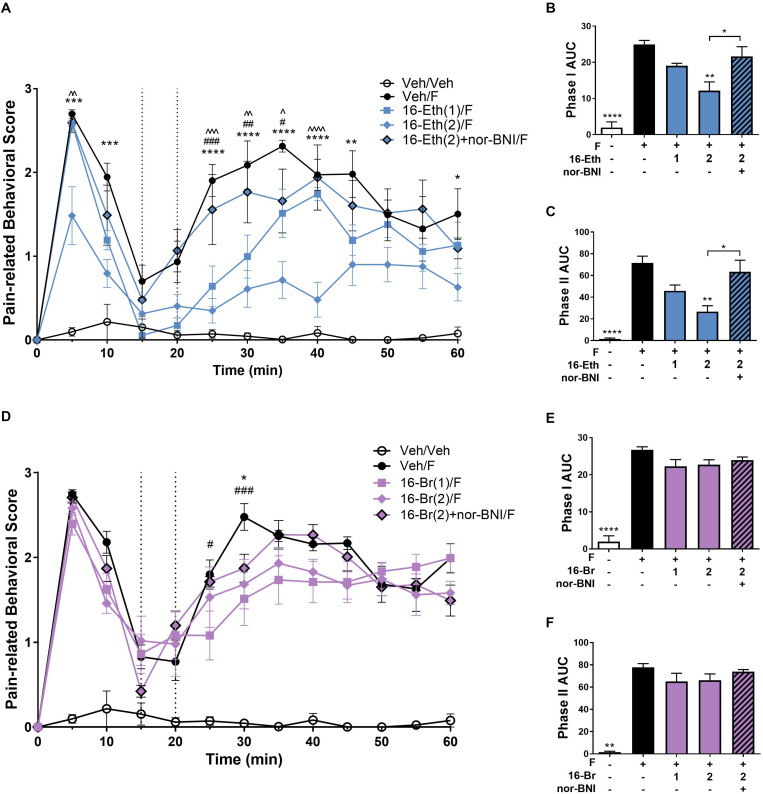
Antinociceptive effect of 16-Ethynyl SalA and 16-Bromo SalA in the intraplantar 2% formaldehyde test. **(A)** Time course of pain-related behavior following intraplantar 2% formaldehyde injection into the right hind paw. 16-Ethynyl SalA (1–2 mg/kg i.p.) treatment showed a significant reduction in pain-related behavior compared to the vehicle/formaldehyde-treated control group. **(B,C)** The area under the curve (AUC) was calculated for phase I nociceptive pain-related behavior (0–15 min, **B**) and phase II inflammatory pain-related behavior (20–60 min, **C**). 16-Ethynyl SalA showed a significant reduction in both phases compared to the vehicle/formaldehyde treatment, which was reversed using the KOPr antagonist nor-binaltorphimine (nor-BNI). **(D)** Time course of pain-related behavior for 16-Bromo SalA (1–2 mg/kg i.p.) treatment showed a significant reduction in pain-related behavior compared to the vehicle/formaldehyde-treated positive control group. **(E,F)** AUC for phase I nociceptive pain-related behavior (0–15 min, **E**) and phase II inflammatory pain-related behavior (20–60 min, **F**). 16-Bromo SalA did not show a significant reduction in both phases of pain-related behavior compared to the vehicle/formaldehyde control group. **(A,D)** Two-way repeated-measures ANOVA with Bonferroni post-tests. *p < 0.05, **p < 0.01, ***p < 0.001, ****p < 0.0001 for 2 mg/kg dose vs. vehicle/formaldehyde control. ^#^*p* < 0.05, ^##^*p* < 0.01, ^###^*p* < 0.001 for 1 mg/kg dose vs. vehicle/formaldehyde control. ^∧^*p* < 0.05, ^∧∧^*p* < 0.01, ^∧∧∧^*p* < 0.001, ^∧∧∧∧^*p* < 0.0001 for comparison between the 2 mg/kg with and without nor-BNI pre-treatment. **(B,C,E,F)** Kruskal–Wallis test with Dunn’s post-tests. Values presented as mean ± SEM, *n* = 6–7 and *n* = 5 for nor-BNI antagonist group. Number in brackets indicates dose in mg/kg and F = formaldehyde i.pl. administration.

In the intraplantar formaldehyde assay, 16-Ethynyl SalA (2 mg/kg) had a significant antinociceptive effect from 5 to 10 min, 25 to 45 min, and 60 min, and the 1 mg/kg dose had a significant effect at 25–35 min compared to the vehicle/formaldehyde treatment (Figure 4A). Area under the curve analysis for phase I and phase II pain-related behavior showed 16-Ethynyl SalA reduced the pain-related behavioral scores at 2 mg/kg but not 1 mg/kg and nor-BNI significantly reversed the effect of the 2 mg/kg dose (Figures 4B,C).

A 16-Bromo SalA was tested via i.p. administration in the intraplantar formaldehyde model. Over the time course, the 2 mg/kg dose of 16-Bromo SalA reduced pain-related behavioral scores at 30 min and the 1 mg/kg dose reduced pain-related behavioral scores at 25 and 30 min (Figure 4D). 16-Bromo SalA did not show a significant reduction in phase I (Figure 4E) or phase II pain-related behavioral scores (Figure 4F) for either dose.

**FIGURE 5:**
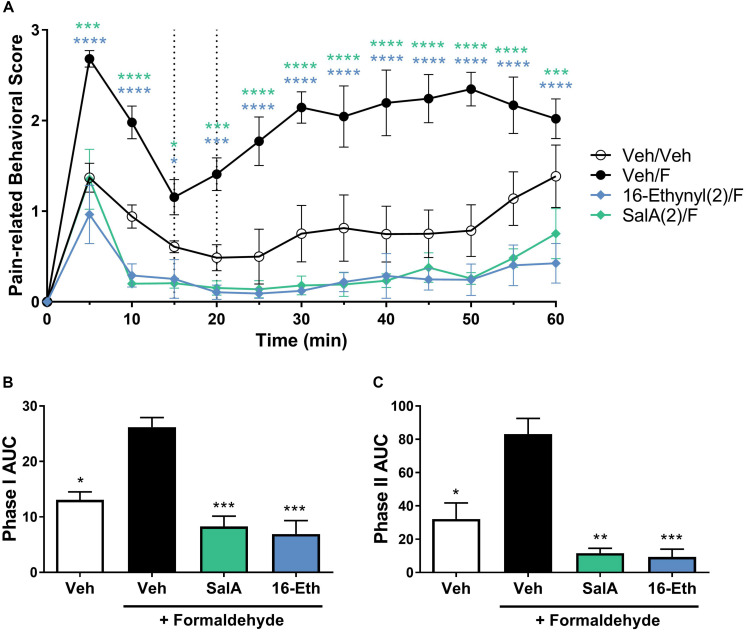
Local adminstration of 16-Ethynyl SalA produced antinocieptive effects in the intraplantar 2% formaldehyde assay. **(A)** Time course of pain-related behavior following intraplantar 2% formaldehyde injection into the right hind paw. 16-Ethynyl SalA (2 mg/kg i.pl.) treatment showed a significant reduction in pain-related behavior compared to the vehicle/formaldehyde-treated control. Two-way repeated-measures ANOVA with Bonferroni post-tests. **(B,C)** Area under the curve (AUC) analysis of phase I **(B)** and II **(C)** pain-related behaviors. 16-Ethynyl SalA reduced both phases of pain-related behavior. Kruskal–Wallis test with Dunn’s post-tests. *p < 0.05, **p < 0.01, ***p < 0.001, ****p < 0.0001 for 2 mg/kg doses vs. vehicle/formaldehyde control. Values presented as mean ± SEM, *n* = 6. Number in brackets indicates dose in mg/kg and F = formaldehyde i.pl. administration.

Due to the significant effects of 16-Ethynyl SalA via i.p. administration in the intraplantar formaldehyde model, the compound was also tested via i.pl. administration. Figure 5 shows that 16-Ethynyl SalA have antinociceptive effects when administered locally within the same paw as 2% formaldehyde. SalA (2 mg/kg i.pl.) and 16-Ethynyl SalA reduced the pain-related behavioral scores between 5 and 60 min. In the area under the curve analysis, SalA and 16-Ethynyl SalA reduced both phases of pain-related behavior (Figures 5B,C).

**FIGURE 6:**
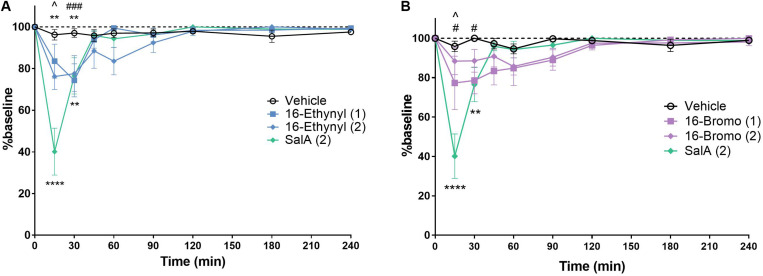
KOPr agonists cause motor incoordination in the rotarod performance assay. Using a rotarod apparatus set to accelerate from 4 to 40 rpm over 300 s, SalA showed a significant effect at 15–30 min. **(A)** 16-Ethynyl showed a significant effect at 15–30 min. **(B)** 16-Bromo SalA had a significant impairment at 15–30 min at 1 mg/kg. Two-way repeated-measures ANOVA, **p < 0.01, ****p < 0.0001 for 2 mg/kg dose (above) or SalA (below) vs. vehicle. ^#^*p* < 0.05, ^###^*p* < 0.001 for 1 mg/kg dose vs. vehicle. ^∧^indicates time point where SalA is more sedative than both doses of the novel analog. Values presented as mean ± SEM, *n* = 6.

The rotarod performance test measures motor coordination and is frequently used to assess the sedative effects of novel drugs. 16-Ethynyl SalA and 16-Bromo SalA were compared to SalA (2 mg/kg i.p.), which is known to cause motor incoordination in the rotarod performance test in mice (Kivell et al., 2018). The time course showed that 16-Ethynyl SalA decreased latency to fall between 15 and 30 min (Figure 6A). At the 15 min time point, SalA was more sedative than both doses of 16-Ethynyl SalA. Similarly, 16-Bromo SalA had sedative effects at 15–30 min at the 1 mg/kg dose only (Figure 6B). Both doses of 16-Bromo SalA were less sedative than SalA at the 15 min time point.

**FIGURE 7:**
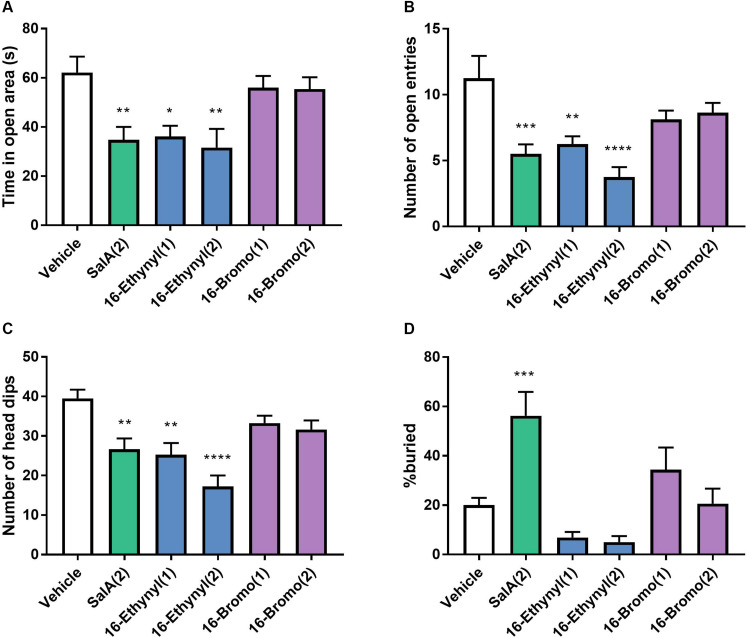
Biased agonist 16-Bromo SalA does not have anxiogenic side effects compared to balanced agonist 16-Ethynyl SalA. Mice were tested on the elevated zero maze for 5 min with recordings made for the **(A)** time in the open area, **(B)** the number of open entries, and **(C)** number of head dips. SalA (2 mg/kg i.p.) and 16-Ethynyl SalA (1–2 mg/kg i.p.) reduced the all the behaviors, whereas, 16-Bromo SalA (1–2 mg/kg i.p.) had no effect. **(D)** The marble-burying experiment showed SalA administration lead to a significant increase in the percentage of marbles buried, whereas, the novel analogs did not alter the marble count. One-way ANOVA with Bonferroni post-tests. *p < 0.05, **p < 0.01, ***p < 0.001, ****p < 0.0001 compared to vehicle control. Values presented as mean ± SEM, *n* = 8.

The anxiogenic effects of 16-Ethynyl SalA and 16-Bromo SalA were evaluated using the elevated zero maze and marble burying tests. SalA (2 mg/kg i.p.) and 16-Ethynyl SalA (1–2 mg/kg i.p.) reduced the time in open area, open arm entries and number of head dips compared to vehicle, whereas, 16-Bromo SalA (1–2 mg/kg i.p.) did not have any effect (Figures 7A–C). Marble burying was used to measure anxiogenic and obsessive compulsive-like behaviors (Figure 7D). Compared to vehicle, the mice administered SalA (2 mg/kg i.p.) had an increase in the percentage of marble buried, whereas 16-Ethynyl SalA (1–2 mg/kg i.p.) and 16-Bromo SalA (1–2 mg/kg i.p.) did not alter the number of marbles buried.

## Discussion

In this study we compared two C-16 analogs of SalA with varying G-protein biased signaling properties. We showed that 16-Bromo SalA displayed G-protein biased signaling at KOPr, whereas, 16-Ethynyl SalA displayed balanced signaling properties at KOPr.

A 16-Bromo SalA has previously been shown to have a similar binding affinity for the KOPr to that of SalA and U50,488 (16-Bromo SalA K_*i*_ = 2.9 ± 0.3 nM; SalA K_*i*_ = 2.5 ± 0.6 nM; U50,488 K_*i*_ = 2.2 ± 0.2 nM; in CHO cells stably expressing hKOPr with [^3^H]diprenorphine as the radioligand), as well as similar potency (16-Bromo SalA EC_50_ = 2.4 ± 0.2 nM; SalA EC_50_ = 2.1 ± 0.6 nM; U50,488 EC_50_ = 2.9 ± 0.2 nM; measured with the [^35^S]GTP-γ-S functional assay in CHO cells stably expressing the hKOPr) (Beguin et al., 2009). In cellular assays (HitHunter^TM^), 16-Ethynyl SalA displayed a 2-fold increase in potency at KOPr compared to 16-Bromo SalA (0.019 ± 0.004 and 0.040 ± 0.010 nM, respectively) and was more potent than SalA (EC_50_ = 0.030 ± 0.004 nM) and U69,593 (EC_50_ = 0.80 ± 0.40 nM) (Riley et al., 2014). In the current study, 16-Ethynyl SalA and 16-Bromo SalA were more potent than U50,488 in inhibiting forskolin-stimulated cAMP accumulation. However, 16-Bromo SalA was less potent in β-arrestin recruitment assays. Thus, 16-Bromo SalA was revealed to be G-protein biased, whereas 16-Ethynyl SalA displayed balanced signaling. It has been hypothesized, that β-arrestin recruitment is responsible for many KOPr-mediated adverse effects (Phillips et al., 2013; Zhou et al., 2013). In support of this, the biased agonist RB-64, showed no sedation, motor incoordination, or anhedonia-like effects in mice (White et al., 2015) and the only clinically available KOPr agonist, nalfurafine, has been shown to be extremely G-protein biased (Schattauer et al., 2017). However, few reports have fully explored biased signaling at KOPr and correlated this to antinociceptive effects and side effects in structurally similar agonists (DiMattio et al., 2015; White et al., 2015; Dunn et al., 2018, 2019; Ho et al., 2018; Kaski et al., 2019; Mores et al., 2019). In addition, a recent paper showed low intrinsic efficacy, rather than G-protein bias, could explain the reduced respiratory side effects in MOPr agonists (Gillis et al., 2020).

To further investigate the properties of the C-16 analogs of SalA, the antinociceptive effects were measured in thermal models of pain. The *in vivo* antinociceptive dose-response effects in the warm water tail-withdrawal assay show that both 16-Ethynyl SalA and 16-Bromo SalA had increased potency compared to U50,488. This effect mirrors the increased potency of the novel analogs in G-protein signaling pathway compared to U50,488.

One of the limitations of SalA is that it is rapidly metabolized to the inactive Salvinorin B, and therefore the antinociceptive effects of SalA are short-acting (Butelman et al., 2009; Teksin et al., 2009; Ranganathan et al., 2012). Because of this known limitation, we evaluated the antinociceptive duration of action of the SalA analogs in warm water tail-withdrawal and hot-plate assays. We have previously shown SalA to have a duration of 30 min in our model (Paton et al., 2017) while McCurdy et al. (2006) found that SalA (2–4 mg/kg) had a duration of action of 20 min in the tail-withdrawal assay and 10 min in the hot-plate (52°C) assay in male Swiss mice. 16-Ethynyl SalA (2 mg/kg) had a longer duration of action with significant antinociception at 5–60 min. 16-Bromo SalA (2 mg/kg) had a slower onset of action, with significant effects at 10–60 min. Similar effects were observed in the hot-plate test with all compounds having antinociceptive effect up to 30 min. In addition, 16-Ethynyl SalA had greater antinociceptive effect than SalA and 16-Bromo SalA at the 30 min time point. The longer duration of action of the novel analogs indicate that these structural modifications have improved pharmacokinetic profiles as these analogs were designed to decrease the metabolism of the furan ring of the SalA scaffold (Riley et al., 2014). C-2 alterations of the SalA structure, such as the analog ethoxymethyl ether (EOM) SalB, are more metabolically stable (Ewald et al., 2017). While less is known about the effects of C-16 alterations, it has been predicted that modifications at the C-16 position may hinder the action of cytochrome P450 enzymes (Wilson et al., 1990). Since the short duration of action of SalA is one factor that has limited the clinical development of SalA analogs agonists, the increase in the duration for the novel analogs is a promising development.

The antinociceptive effects of the KOPr agonists were further assessed using the intraplantar formaldehyde model of both nociceptive and inflammatory pain. The i.p. administration of 16-Ethynyl SalA produced antinociceptive effects in phase I at 2 mg/kg and in phase II at both 1 and 2 mg/kg doses, which was prevented by administration of the KOPr antagonist nor-BNI. 16-Bromo SalA did not reduce phase I nor phase II overall, however, did have a significant effect at the 25–30 min time point when time-course data was analyzed. Aviello et al. (2011) hypothesized that higher doses of SalA (2 mg/kg) may have a central target, whilst lower doses (0.5 mg/kg) appeared to have a peripheral effect greater than the central effects. In the present study, 16-Ethynyl SalA was also administered via i.pl. injection at the site of inflammation, to assess local effects. At 2 mg/kg SalA reduced pain-related behavioral scores and reduced both phase I and II pain-related behavioral scores following AUC analysis. Similarly, 16-Ethynyl SalA was effective at reducing both phases of pain-related behavior. Overall, 16-Ethynyl SalA was shown to be more effective at reducing nociceptive and inflammatory pain-related behavior. However, it would be worth further exploring the effects of these compounds in pain-depressed behavioral models, such as models that measure a decrease in the level of feeding or locomotor activity when animals are in a pain-like state (Negus et al., 2010). These models avoid the false positive outcomes that can occur in pain-stimulated behavioral models when drugs have sedative effects (Negus, 2019).

Kappa opioid receptor agonists, including SalA have previously been shown to have sedative effects. White et al. (2015) found that SalA induced motor incoordination in the rotarod performance test for up to 30 min at doses between 3 and 10 mg/kg in both female and male C57BL/6J mice, whereas the G-protein biased agonist, RB-64, did not cause any motor deficits. In male NIH Swiss mice, SalA (0.5–2 mg/kg) disrupted climbing behavior in the inverted screen task, a model of motor coordination, with the effect lasting only 5 min, compared to U69,593 (1 mg/kg) for which the effects lasted 10 min (Fantegrossi et al., 2005). We have previously shown in Sprague–Dawley rats, that 16-Bromo SalA (1 mg/kg) and 16-Ethynyl SalA (0.3–2 mg/kg) did not have an effect on spontaneous locomotor activity (Riley et al., 2014; Mathew, 2019). In the present study, the effects of the KOPr agonists on motor coordination were measured over 240 min, and SalA, 16-Ethynyl SalA and 16-Bromo SalA were found to have sedative effects for up to 30 min. SalA had a longer duration than we have previously reported (Kivell et al., 2018); however, the previous study used a rotarod at a set-speed (16 rpm), whereas the current study used an accelerating procedure, which better measures motor coordination (Deacon, 2013). Interestingly, at the 15 min time point, SalA was more sedative than both 16-Ethynyl SalA and 16-Bromo SalA, showing the novel analogs have an improvement over SalA.

Salvinorin A has anxiogenic side effects mediated by the KOPr system (Braida et al., 2009), and in particular, we have previously shown that SalA has anxiogenic effects in the elevated plus maze in rats (Ewald et al., 2017). In the current study, we chose to test two models of anxiety in mice to get a better understanding of this complex side-effect. The elevated zero maze measures the conflict between exploratory behaviors and the avoidance of open and elevated areas (Shepherd et al., 1994), whereas, the marble burying test measures a repetitive, compulsive behavior triggered by anxiety (Broekkamp et al., 1986). In the elevated zero maze, SalA reduced the time in the open area, the number of open entries and number of head dips with 16-Ethynyl SalA having similar effects, whereas, 16-Bromo SalA had no effect. Similarly, the marble-burying experiment showed SalA administration lead to a significant increase in the percentage of marbles buried, while the novel analogs did not alter the marble count. It is important to consider the effect of locomotor activity, as the motor incoordination could indicate that the mice would have impaired locomotor activity. However, this does not appear to be the case in the marble burying task, as SalA, which produced significant motor incoordination compared to the novel analogs, lead to an increase in the number of marbles buried. Overall, 16-Bromo SalA but not 16-Ethynyl SalA had no observed anxiogenic side effects, which is an improvement over SalA, and does correlate with the theory of G-protein bias agonists having fewer side effects. However, 16-Bromo SalA was also less potent in the pain-related behavioral models, which could explain a lack of potency in the side effects also. Further work is warranted to fully understand any other KOPr-mediated side effects such as aversive and depressive-like effects.

Overall, 16-Ethynyl SalA had significant antinociceptive effects in acute nociceptive and inflammatory pain models and had reduced side effects compared to the parent compound SalA. 16-Bromo SalA reduced some pain-related behaviors and did not show any anxiogenic side effects.

## Data Availability Statement

The raw data supporting the conclusions of this article will be made available by the authors, without undue reservation.

## Ethics Statement

The animal study was reviewed and approved by the Victoria University of Wellington Animal Ethics Committee (approval numbers 21480 and 25751).

## Author Contributions

BK, TP, AL, and KP contributed to the design of the study. TP, SK, and RC provided the kappa opioid receptor agonists. KP, AB, SK, and RC conducted the experiments and performed the data analysis. KP wrote the first draft of the manuscript. BK, TP, and AL critically evaluated the manuscript. All authors contributed to the manuscript revision, read, and approved the submitted version.

## Conflict of Interest

The authors declare that the research was conducted in the absence of any commercial or financial relationships that could be construed as a potential conflict of interest.
